# Association between temporomandibular disorders and somatization: a narrative review

**DOI:** 10.22514/jofph.2026.004

**Published:** 2026-01-12

**Authors:** Qing Xue, Hongyu Ming, Yi Huang, Xin Xiong

**Affiliations:** ^1^Department of Stomatology, The Second Affiliated Hospital of Shandong First Medical University, 271000 Tai’an, Shandong, China; ^2^State Key Laboratory of Oral Diseases, National Centre for Stomatology, and National Clinical Research Centre for Oral Diseases, West China Hospital of Stomatology, Sichuan University, 610041 Chengdu, Sichuan, China; ^3^Centre for Research and Development, Pan-European University, 821 02 Bratislava, Slovakia

**Keywords:** Temporomandibular disorders, Somatization, Orofacial pain, Biopsychosocial

## Abstract

Temporomandibular disorders (TMD) are common orofacial pain conditions with multifactorial etiologies. Somatization refers to the manifestation of psychological distress as physical symptoms in the absence of clear medical causes. A growing body of clinical research has recently shown a strong association between TMD and somatization. A substantial proportion of TMD patients exhibit moderate to high levels of somatic symptoms, leading to greater pain intensity, longer disease duration, and heightened psychological distress. The TMD-somatization relationship has been underpinned by complex pathophysiological interactions and the underlying mechanisms involved, including central sensitization (CS), potential biomarkers, nociplastic pain, neurobiological changes, and so on. Clinically, recognizing somatization in TMD patients is essential, as it can adversely affect treatment outcomes and necessitate a biopsychosocial management approach. In this narrative review, we summarize the clinical evidence of the TMD-somatization association, discuss the underlying mechanisms, explore management implications, and identify directions for future research.

## 1. Introduction

Temporomandibular disorders (TMD) encompass a group of musculoskeletal conditions affecting the temporomandibular joint (TMJ), masticatory muscles, and associated structures, which are characterized by jaw pain, functional limitations, TMJ sounds, and sometimes joint locking episodes [1, 2]. Recent systematic reviews estimated a high global prevalence rate of TMDs, ranging from 31% to 34%, with females exhibiting a higher prevalence than males [3, 4, 5]. Clinically, TMD presents with jaw or orofacial pain, muscle tenderness, joint sounds, limited mouth opening, and headaches [6, 7]. The pathogenesis of TMD is considered multifaceted, involving an interplay of biological factors, biomechanical factors, neuromuscular factors, psychological factors, and social factors [8]. The clinical diagnosis of TMD is commonly based on the Diagnostic Criteria for TMD (DC/TMD), which has been proven reliable and valid. It provides a formal dual-axis framework for diagnosis, classifying TMD into pain-related and intra-articular subtypes, while also assessing psychosocial factors on a separate axis. Specifically, Axis I identifies physical diagnoses via clinical exams, while Axis II evaluates psychosocial impacts [9]. Clinical exams confirm TMD subtypes and differentiate them from other orofacial pain conditions. Modern diagnostics like the DC/TMD emphasize evaluating both physical and psychological aspects [10, 11]. This means that successful management requires addressing not only the peripheral anatomical issues but also the contributing behavioral and psychosocial elements [12, 13].

Somatization is the expression of psychological distress through physical symptoms, such as pain, fatigue, and gastrointestinal issues, without clear medical causes [14]. Classified as Somatic Symptom Disorder in the Diagnostic and Statistical Manual of Mental Disorders, Fifth Edition (DSM-5), it emphasizes both symptoms and excessive health anxiety. Moreover, somatization correlates strongly with anxiety and depression. Assessment tools for somatization include the Patient Health Questionnaire-15 (PHQ-15) and Somatic Symptom Scale-8 (SSS-8), both effective in quantifying symptom burden [15, 16], and the DC/TMD Axis II protocol uses the PHQ-15 to assess somatic symptom severity. The Physical Symptom Scale-8 (PSS-8), tailored for TMD, adapts the SSS-8 with modified scoring. Tools like the Symptom Checklist-90-Revised (SCL-90-R) somatization subscale historically evaluated symptom preoccupation, while the Research Diagnostic Criteria for TMD (RDC/TMD) differentiated TMD-related symptoms from broader complaints [17].

Somatization is of particular clinical importance in TMD. Many TMD patients present with diffuse bodily complaints, such as headaches, muscle aches, and gastrointestinal discomfort, reflecting a somatic expression of stress or emotional turmoil [18, 19]. Given the potential for somatization to exacerbate pain perception, complicate diagnosis, and hinder standard TMD treatments, a thorough review of the TMD-somatization association is warranted. This review was conducted to summarize the association between TMD and somatization based on the clinical evidence, discuss the mechanisms linking them, and explore the implications for management, along with future research directions. Understanding this interplay is essential for clinicians to provide effective, holistic care for TMD patients.

## 2. Methods

### 2.1 Search strategy

Relevant literature was identified through searches in PubMed, Embase, and Web of Science. Keywords used in the search included “temporomandibular disorders”, “temporomandibular joint”, “somatization OR somatic”, “orofacial pain”, and “biopsychosocial”.

### 2.2 Inclusion and exclusion criteria

Articles were included if they: (1) focused on TMD therapy related to somatization; (2) discussed the association between TMD and somatization. Articles were included if they met any of the criteria.

### 2.3 Study selection

Three reviewers independently screened titles and abstracts for relevance. Full-text articles were evaluated to confirm they met the inclusion criteria. Disagreements were resolved through discussion to reach a consensus.

### 2.4 Data extraction and synthesis

Findings were organized thematically into related mechanisms, clinical implications, management strategies, research gaps, and future directions to ensure a comprehensive and structured analysis.

## 3. The association between TMD and somatization

### 3.1 Prevalence of somatization in TMD

A growing body of clinical research has documented a robust association between TMD and somatization. Evidence from epidemiological surveys, cross-sectional clinical studies, and intervention trials consistently shows that TMD patients frequently exhibit somatic symptom burdens that exceed general population norms. A retrospective study of East Asian patients seeking TMD care found that 52% had clinically relevant somatization as measured by the PHQ-15, and other reports indicate that approximately 28%–77% of TMD patients experience moderate to severe somatic symptoms [20]. By contrast, individuals without TMD tend to have much lower somatic symptom scores. A recent study reported that all Depression-Anxiety-Stress Scale (DASS-21) measures—and even the “fear of missing out (FOMO)” score—were significantly higher in patients with somatization and orofacial pain compared to pain-free controls [21]. Health anxiety, classified as a somatic symptom and related disorder in the DSM-5, has been reported in approximately one-fifth of TMD patients, with a higher prevalence observed in those with pain-related TMDs [22, 23]. These findings suggest that somatization is a common comorbidity among individuals with TMD.

### 3.2 Somatic symptom severity and functional impact

Building on these prevalence data, numerous studies demonstrate that somatization in TMD patients is associated with more severe and persistent symptoms. Patients with higher somatic symptom burdens typically report increased pain intensity, greater jaw disability, and longer pain duration. Studies differentiating TMD patients by somatization status reveal that those with significant somatic symptoms endure longer-lasting pain and greater interference with daily activities compared to those without [24, 25]. For example, pain-related disability, such as difficulties in work or social activities due to jaw pain, is notably higher when somatization co-occurs [26, 27]. Furthermore, one investigation found a weak but positive correlation between chronic masticatory muscle pain (assessed by both palpation and patient report) and somatic symptom severity (as measured by SSS-8 scores) [28]. Similarly, composite measures of chronic pain severity, which combine pain intensity and life interference, also show a weak yet significant correlation with somatic symptom levels. Collectively, these findings suggest that TMD patients burdened by widespread somatic symptoms tend to experience the most severe pain and functional impairment, indicating a mutually reinforcing relationship between localized pain and general somatization.

### 3.3 Psychological distress and migraine

Much evidence shows that TMD patients with high somatization also exhibit elevated psychological distress [29, 30, 31]. Interestingly, a recent study has found that somatization had a strong association with depression and anxiety, whereas facial pain intensity was less strongly correlated with these psychological measures in TMD patients [20]. This further suggests that somatization may serve as a marker for the psychosocial domain of TMD rather than being directly tied to peripheral pain.

Migraine is a common neurobiological headache disorder, and many studies have revealed high comorbidity and a clear association between TMD and migraine. A study comparing TMD patients with and without migraine found that those with migraine, who reported more widespread symptoms, scored significantly higher on measures of somatization, depression, and anxiety, indicating that a higher somatic symptom burden in TMD is part of a broader pattern of psychological distress and is associated with comorbid migraine [32]. Migraine may act as an independent or additive factor contributing to both somatic symptoms and psychological distress. Moreover, TMD pain and migraine appear to be associated with different psychological distress. To be specific, situational anxiety coupled with a lack of coping strategies may be more strongly associated with TMD pain, while trait anxiety and depression might be more closely linked to migraine [33]. In general, there are complex relationships among TMD, somatization, migraine, and psychological distress, which need to be further explored in future research.

### 3.4 Impact on treatment outcomes

The level of somatization may influence treatment outcomes in TMD. Some studies have shown that elevated somatization is a predictor of poor TMD treatment outcome, linking high somatization to poorer self-reported improvement and satisfaction with TMD treatment [20, 34]. Notably, a brief cognitive-behavioral therapy (CBT) trial for chronic TMD pain found that patients with high somatization benefited less from the intervention compared to those with low somatization. Low-somatized patients experienced greater pain reduction over time with CBT, whereas high-somatized patients continued to report persistent pain. This suggests that somatization was a significant moderator of TMD treatment effects on pain-related interference with functioning [35]. Importantly, current evidence remains limited, and higher somatic burden does not uniformly imply poorer prognosis. Instead, somatization functions as a complex treatment moderator requiring dialectical interpretation and individualized therapeutic approaches rather than deterministic outcome predictions.

### 3.5 Bidirectional relationships and clinical implications

Emerging research hints at bidirectional relationships between TMD and somatization. On one hand, a high somatic symptom burden may predispose individuals to develop TMD or exacerbate an existing condition [36]. For instance, a cross-sectional study of young adults found that those with higher somatization scores had greater odds of reporting TMD pain, even after adjusting for emotional factors. This implies that general bodily distress may lower the threshold for perceiving jaw-related pain or prompt individuals to seek treatment [12]. On the other hand, chronic TMD pain can itself lead to somatization over time. Chronic pain is known to induce hypervigilance to bodily sensations, and TMD may trigger broader health anxiety or increased symptom awareness [37]. The same study identified that being female, having TMD, and exhibiting high negative affectivity were significant risk factors for somatization in young adults [38, 39]. Essentially, TMD and somatization likely cyclically reinforce each other, making it challenging to untangle cause and effect.

Preliminary evidence indicates that interventions targeting the psychosomatic aspects of TMD can alleviate somatization. A recent pilot randomized controlled trial evaluated a social media-based Pain Neuroscience Education (PNE) program combined with self-management for young adults with TMD [40]. Following a one-week intervention featuring animated educational videos about pain mechanisms and stress management techniques, the group receiving PNE alongside standard self-care advice showed a significant reduction in pain somatization scores compared to the control group that received self-care advice alone. The reduction in somatization suggests that educating patients about the mind-body connection can lessen their somatic symptom burden. However, it is important to recognize that, as a pilot study, the experiment did not show statistically significant changes in pain intensity, and the conclusions drawn remain preliminary and exploratory in nature. Other therapies, such as mindfulness-based stress reduction and multidisciplinary rehabilitation, have shown promise in analogous chronic pain conditions and are being explored in TMD with the expectation that they may similarly reduce somatic distress [41, 42, 43, 44].

In summary, clinical evidence robustly supports an association between TMD and somatization. TMD patients commonly exhibit elevated somatic symptoms, which correlate with more severe pain and psychological distress, and this interplay likely complicates treatment while also offering avenues for therapeutic intervention. Recognizing this association, clinicians should assess somatic symptom burden as part of a comprehensive evaluation of TMD patients. Addressing somatization may be as crucial as treating the TMJ or muscular pathology itself, given its significant impact on the overall disease experience.

## 4. Pathophysiological mechanisms connecting TMD and somatization

The association between TMD and somatization has been underpinned by complex pathophysiological interactions, mainly involving the nervous system’s processing of pain and stress. Several mechanisms have been proposed to explain why patients with TMD pain often have heightened somatic symptom reporting.

### 4.1 Central sensitization

Central sensitization (CS) is defined as an increased responsiveness of neurons within the central nervous system to sensory stimulation, resulting in pain hypersensitivity [45]. Chronic TMD pain, particularly myofascial pain, is thought to involve CS, similar to what is observed in other functional pain syndromes such as fibromyalgia, irritable bowel syndrome, and chronic headaches [46]. In these conditions, the nervous system becomes hyperexcitable so that normal stimuli produce exaggerated pain responses (allodynia or hyperalgesia), and pain may even spread beyond its original location [47]. To be specific, the mechanisms that induce hyperalgesia mainly include C-fiber-limbic system crosstalk and neuroplasticity in stress-induced hyperalgesia. Many studies demonstrated that C-fibers convey nociceptive signals to limbic structures, which are hyperresponsive in stressed mice. This pathway underlies the affective-motivational dimension of pain and aligns with clinical findings in TMD patients with somatic symptoms [48, 49]. Moreover, Social defeat stress (SDS) models revealed that hyperalgesia is mediated by Nav1.8 C-fibers and central cholecystokininergic systems, which are also implicated in depression [50]. SDS simultaneously sensitizes peripheral nociceptors and heightens limbic reactivity, creating a vicious cycle [51].

These mechanisms may also account for somatization, as individuals with CS tend not only to experience amplified jaw pain but also to perceive other bodily sensations more intensely [1]. Indeed, research indicates that TMD patients with higher somatic symptom burdens often exhibit widespread hyperalgesia and score higher on the Central Sensitization Inventory (CSI) [52]. One observational study reported a weak yet significant correlation between somatization and CSI scores in TMD patients, with those exhibiting clinically relevant CS tending to report more severe somatic symptoms [53]. Ultimately, the central nervous system amplification underlying CS may manifest as both intensified pain and a range of other physical symptoms.

### 4.2 Oxidative stress biomarkers

Oxidative stress—defined as an imbalance between reactive oxygen/nitrogen species and antioxidants—has been implicated as a contributor to both chronic pain and depression [54]. In patients with TMD myofascial pain, levels of certain oxidative stress biomarkers have been associated with the severity of somatic symptoms [55]. Recent studies have begun to examine biochemical markers in TMD patients in order to identify objective correlates of pain chronicity and psychosocial burden [56, 57]. For example, one study measured salivary and blood concentrations of various oxidative/nitrosative stress markers, such as malondialdehyde (MDA), lipid hydroperoxides (LOOH), and antioxidant levels, alongside psychosocial questionnaires [58]. It found that higher somatization scores, as measured by the PHQ-15, were associated with alterations in these biomarkers. Specifically, patients with severe somatization exhibited different oxidative marker profiles compared to those with minimal symptoms [59]. These findings suggest that oxidative stress may act as a physiological “co-player” in TMD patients with a high somatic burden, although causation remains unestablished. It is possible that systemic oxidative stress contributes to muscle fatigue and pain, thereby amplifying somatic complaints, or conversely, that the burden of chronic pain and poor sleep in TMD patients elevates oxidative stress levels.

### 4.3 The vicious cycle mechanism

Anxiety plays a central role in exacerbating TMD pain through a vicious cycle involving somatization [60]. Psychological distress, especially anxiety, heightens somatic awareness, which is a strong predictor of TMD onset and chronicity. This heightened somatic focus amplifies pain perception and promotes maladaptive behaviors such as jaw muscle hyperactivity and avoidance of normal jaw functions, which further strain the TMJ and masticatory muscles, worsening pain symptoms. In turn, persistent pain and somatic symptoms increase anxiety and emotional distress, reinforcing this self-perpetuating cycle. Neurobiological changes, including CS and neuroendocrine dysregulation, lower pain thresholds, and intensify somatic symptom reporting, making benign sensations feel threatening and fueling somatization. This interplay complicates diagnosis and treatment, as patients often present with overlapping psychological and physical symptoms that maintain and amplify each other [61]. Clinically, this vicious cycle underscores the importance of addressing both psychological and somatic components in TMD management (Fig.1). Understanding and targeting the anxiety-somatization-pain loop is essential for effective treatment and prevention of chronic TMD [62].

**Fig. 1.** The vicious cycle mechanism on the association between TMD and somatization. Anxiety plays a central role in exacerbating TMD pain through a vicious cycle involving somatization. TMD, temporomandibular disorders.

### 4.4 Findings from brain imaging

Although direct neuroimaging evidence linking somatization to TMD is limited, pain-related functional magnetic resonance imaging (fMRI) studies provide valuable insights. Chronic TMD patients have demonstrated altered functional connectivity within pain-related brain networks and changes in gray matter volume in regions such as the prefrontal cortex, an area crucial for pain modulation and emotional regulation [63, 64]. In patients with concomitant somatic symptoms, even broader alterations in brain networks related to interoception may be expected [65]. The insula, a region that integrates bodily sensations with emotional context, is frequently activated in both TMD pain and somatic symptom disorders [66]. Enhanced insular activity may explain why some TMD patients experience widespread discomfort, as their brains appear to be overly attuned to various bodily signals [67]. Similarly, the anterior cingulate cortex, which processes the affective component of pain, may remain hyperactive in somatized patients, contributing to both persistent pain and general malaise.

### 4.5 Nociplastic pain and neuroplastic changes

The International Association for the Study of Pain (IASP) defines nociplastic pain as pain arising from altered nociception without clear evidence of tissue injury or nerve damage, often accompanied by hypersensitivity [68]. Many patients with chronic, painful temporomandibular disorder (TMD) meet this definition after the exclusion of ongoing inflammation or neuropathy [69]. Proposed criteria for nociplastic TMD pain include chronic regional pain with hypersensitivity to pressure or movement in the jaw, as well as comorbid symptoms such as sleep disturbances and fatigue. These features overlap with somatization, given that such symptoms are commonly captured in somatic symptom scales. Neuroimaging studies of TMD and other chronic pain patients have identified both functional and structural brain changes, including hyperactivity in regions such as the insula and anterior cingulate cortex—areas associated with the affective processing of pain and interoception—and disruptions in pain modulation circuits [70, 71]. These neural alterations may increase an individual’s vigilance to bodily sensations, leading to a heightened perception of discomfort or pain. Consequently, in TMD patients with high somatic awareness, it is plausible that a neurobiological predisposition—characterized by an altered “pain matrix”—heightens both jaw pain and other non-TMD somatic symptoms.

### 4.6 Psychological and cultural factors

Somatization can also be understood from a psychological perspective as a defense mechanism. Individuals who, due to personal or cultural factors, are reluctant to express anxiety or depression may unconsciously convert emotional distress into physical symptoms [72]. This process does not create pain out of thin air; rather, it magnifies pre-existing sensations. In the context of TMD, patients who subconsciously rely on somatic symptoms as a coping strategy may focus excessively on jaw pain and even report new symptoms as their stressors evolve [73, 74]. Moreover, fear and catastrophic thinking about these symptoms can further exacerbate the cycle [75]. For instance, a patient might misinterpret benign jaw noises or mild discomfort as signs of a serious condition, which then triggers anxiety, increases muscle tension, and heightens pain perception. This vicious cycle of hypervigilance and fear towards bodily sensations is a recognized pathway in chronic pain and somatic symptom disorders.

### 4.7 Psychoneuroimmunological pathways

Chronic stress and psychological distress, which frequently accompany TMD pain and somatic symptom disorder, can disrupt the hypothalamic-pituitary-adrenal (HPA) axis and sympathetic nervous system [76]. This disruption leads to elevated levels of pro-inflammatory cytokines and neurotransmitters that facilitate pain [77]. In stressed TMD patients, increased cortisol or adrenaline levels further heighten muscle tension and pain sensitivity. Over time, such neuroendocrine activation may contribute to CS and produce the systemic symptoms that patients report. Moreover, chronic pain itself acts as an additional stressor, perpetuating a vicious cycle of heightened vigilance and symptom amplification [78].

In conclusion, the mechanisms linking TMD and somatization are multifactorial. CS provides a unifying biological explanation by amplifying both local pain and widespread somatic symptoms. This central mechanism is further modulated by psychological factors, biochemical alterations, and cultural conditioning, creating a scenario in which TMD patients experience not only jaw pain but also a constellation of other bodily symptoms. Understanding these interconnected pathways underscores the need for comprehensive management strategies that address both the peripheral pathology and central neural dysfunction in TMD.

## 5. Clinical implications and management strategies

The strong link between TMD and somatization has several practical implications for patient management. Based on the biopsychosocial approach, we outline key strategies and considerations for managing TMD patients with a high somatic symptom burden.

### 5.1 Comprehensive assessment

Clinicians should routinely screen for somatic symptoms and psychosocial factors when evaluating TMD patients. Standardized tools such as the PHQ-15 and the SSS-8 can help identify individuals with elevated somatization [79]. In practice, even a few targeted questions about unexplained symptoms—for example, asking whether a patient often experiences other pains or physical symptoms in addition to jaw pain—can facilitate open dialogue. When a patient reports widespread or chronic symptoms such as fatigue, headaches, or gastrointestinal issues, it signals the need to address these issues within the overall care plan [80]. Early recognition of somatization can also help temper unrealistic expectations. For example, a single dental procedure is unlikely to resolve a patient’s overall symptom burden if that burden originates from central pain processing.

### 5.2 Pharmacological treatments

When somatization and TMD are prominent, pharmacotherapy can be used to temporarily modulate the nervous system’s response, thereby creating a window of opportunity. During this period of reduced pain, patients are better positioned to adopt essential physical and behavioral habit modifications. Pharmacologic agents typically target specific aspects of dysregulation. For example, low-dose tricyclic antidepressants such as amitriptyline or nortriptyline are frequently prescribed for chronic TMD and fibromyalgia due to their pain-modulating and sleep-enhancing effects [81, 82]. These medications help reduce muscle pain and headaches, and by improving sleep quality, they may also lessen daytime somatic complaints. Additionally, serotonin-norepinephrine reuptake inhibitors such as duloxetine or venlafaxine not only address comorbid depression and anxiety but also provide analgesic benefits for centrally mediated pain [83]. For patients with significant health-related anxiety, selective serotonin reuptake inhibitors or anxiolytic therapies may be indicated [84, 85]. For sleep disturbances, which are common in patients with high somatic symptom burdens, improving sleep hygiene or using low-dose trazodone can be beneficial [86]. Crucially, all pharmacologic treatments should be individualized, closely monitored, and framed within the understanding that their primary role is to enable and support the patient’s active engagement in sustained self-management. Therefore, treatment success fundamentally relies on the patient embracing responsibility during the therapeutic window to establish lasting changes, avoiding long-term dependence due to adverse effects.

### 5.3 Physical exercise

In recent years, the positive effects of physical exercise in the treatment of TMD have been widely recognized [87, 88]. Physical exercise exerts potent antioxidative actions in TMD by boosting systemic and local antioxidant defenses—elevating total antioxidant capacity and scavenging free radicals—to counteract lipid peroxidation and downregulate oxidative biomarkers linked to pain severity [89, 90]. Simultaneously, muscle contractions evoke a characteristic cytokine response: an acute surge of interleukin-6 (IL-6) that triggers downstream release of anti-inflammatory mediators while suppressing tumor necrosis factor-alpha (TNF-α) and interleukin-1 beta (IL-1β), thereby attenuating the neuroinflammatory cascade underlying CS [91]. Moreover, combining therapeutic and aerobic exercise induces endogenous opioid peptide release at peripheral, spinal, and supraspinal sites, enhancing descending inhibitory control and producing robust hypoalgesia, manifested as marked reductions in masticatory muscle pain and gains in bite force in multimodal regimens [92]. Above all, we conclude that physical exercise is a non-pharmacological intervention that might reverse CS and has a good therapeutic effect on TMD.

### 5.4 Interdisciplinary management

Patients with a high somatic symptom burden often benefit from an interdisciplinary approach [93]. In addition to the dentist or TMD specialist addressing the jaw-related components, a collaborative team approach is advisable. Involvement of pain psychology or psychotherapy is particularly valuable. CBT tailored to chronic pain can teach coping skills, relaxation techniques, and cognitive reframing to address health anxieties [94]. Although brief CBT trials have shown mixed results in high-somatizing TMD patients, extended CBT or combination therapies may still reduce distress and improve function. Physiotherapy that emphasizes gentle jaw exercises, posture correction, and self-massage can help patients manage muscle tension and reduce pain triggers [88, 95]. Some physiotherapists incorporate relaxation training into their regimen [96]. In complex cases, interdisciplinary pain programs—where patients consult with a dentist, physical therapist, psychologist, and sometimes a physician for medications—offer the most comprehensive care by addressing both the physical and psychosocial aspects concurrently [35].

### 5.5 Patient education and reassurance

Fundamentally, patient education should center on neuroplasticity—explaining how daily habits modulate the nervous system. Monitoring physical activity, nutrition, and sleep is essential, as these factors directly impact neuroplastic processes [97, 98]. Therefore, the patient’s active engagement in treatment is paramount to fostering adaptive changes. Moreover, a cornerstone of effective management is helping patients understand the nature of their condition. Pain Neuroscience Education has demonstrated both feasibility and benefit in TMD care and clinicians should explain how stress and a sensitive nervous system can amplify pain and other physical symptoms [8, 99]. It is important to validate the patient’s experience by emphasizing that their symptoms are real, even though they are being amplified by a hyperresponsive nervous system.At the same time, patients should be reassured that the absence of severe organic disease, as confirmed through appropriate evaluation, is positive news and that TMD, while challenging, is often benign and can improve with proper self-management and therapy [100, 101]. Educating patients that somatic symptoms often fluctuate over time may help reduce catastrophic thinking and, in turn, decrease symptom intensity [102].

### 5.6 Addressing comorbid conditions

TMD patients with somatization may also present with comorbid conditions such as fibromyalgia, irritable bowel syndrome, or chronic fatigue syndrome [103, 104]. These conditions should be acknowledged and managed concurrently, often in collaboration with the patient’s primary care physician or relevant specialists. Treating these comorbidities, such as employing specific medications or therapies for fibromyalgia, can indirectly alleviate TMD symptoms by reducing the overall somatic burden [105]. Similarly, untreated depression or anxiety in TMD patients can exacerbate pain perception and heighten somatic focus [106]. Therefore, referral to mental health providers for appropriate therapy or medication is often essential. In essence, the approach should be to treat the whole patient, not just the jaw.

In general, the management of TMD patients with somatization should be multi-pronged. By combining dental/orofacial treatments with psychosocial care, clinicians can address the pain from both peripheral and central sources. Studies have shown that such comprehensive approaches are more likely to yield pain reduction and improve quality of life. The challenges in these patients are real—they may progress more slowly and need more support—but with patience and an interdisciplinary strategy, even those with a high somatic symptom burden can achieve meaningful relief and improved function.

## 6. Research gaps and future directions

While our understanding of the connection between TMD and somatization has grown significantly, important questions remain. Addressing these research gaps could advance clinical care and improve patient outcomes.

### 6.1 Longitudinal and causal research

Most current evidence comes from cross-sectional studies, which limit the ability to infer cause-and-effect relationships. Long-term cohort studies that follow individuals with elevated somatic symptoms but without TMD could help determine whether they are at increased risk for developing the disorder. Conversely, tracking newly diagnosed TMD patients over time could reveal whether those with higher levels of somatization at baseline experience distinct pain trajectories or poorer outcomes. Clarifying the temporal relationship between somatization and TMD would enhance understanding of risk factors and support the development of early intervention strategies targeting psychosocial vulnerabilities.

### 6.2 Neurobiological studies

Emerging technologies in neuroimaging and neurophysiology provide promising avenues for exploring the brain mechanisms underlying TMD in highly somatized individuals. fMRI and diffusion tensor imaging could help detect alterations in brain connectivity or activity patterns specific to this subgroup [107]. Investigating neurochemical differences, such as variations in neurotransmitter systems, may offer potential biomarkers for diagnosis or prognosis. Additionally, examining genetic and epigenetic profiles, such as polymorphisms affecting serotonin or catecholamine pathways, could shed light on biological predispositions that link somatization with CS and pain modulation.

### 6.3 Biomarker discovery

Preliminary findings on oxidative and nitrosative stress markers point to a potential role for peripheral biomarkers in capturing central pain amplification and somatic symptom burden [108, 109]. Future research should broaden the biomarker panel to include inflammatory cytokines, salivary cortisol as a stress indicator, and other biological signals associated with altered pain processing. Identifying reliable biomarkers would allow for more accurate patient phenotyping and the ability to monitor responses to treatment more objectively. Ultimately, biomarker-guided approaches could personalize care for TMD patients with prominent somatization.

### 6.4 Intervention trials targeting somatization

There is a pressing need for more randomized controlled trials that specifically evaluate treatments aimed at reducing somatization in TMD patients. For example, trials implementing collaborative care models—where a psychologist or psychiatrist is integrated into the TMD treatment team—could determine whether such approaches yield superior outcomes compared to standard care. Similarly, investigating targeted therapies such as mindfulness-based stress reduction, acceptance and commitment therapy, or somatic experiencing in TMD populations may provide evidence for novel treatment strategies. Given the mixed results observed with brief CBT in patients with high levels of somatization, future interventions might consider longer-duration therapy or a combination of treatment modalities. Additionally, pain neuroscience education presents another promising avenue. Large-scale studies are needed to verify its efficacy in reducing not only pain but also the cognitive and somatic dimensions of TMD over the long term.

### 6.5 Integration of care and education

On a systemic level, incorporating education on somatization and chronic pain into both dental and medical training is essential. Many dentists and physicians may not feel adequately prepared to address the psychosocial aspects of TMD. Future efforts could focus on developing clinical guidelines and continuing education programs that equip TMD practitioners with the skills necessary to screen for mental health issues and collaborate effectively with mental health professionals. Moreover, creating patient education materials that specifically address the relationship between TMD, stress, and somatic symptoms can empower patients to participate actively in their care. For instance, a smartphone application for TMD self-management—featuring relaxation recordings, cognitive strategies, symptom tracking, and even direct communication with providers—could merge technological innovation with holistic care and be evaluated in future efficacy studies.

### 6.6 Cultural and demographic considerations

Future research should also explore how the relationship between TMD and somatization varies across different cultures, age groups, and genders. For example, it remains unclear why some Asian populations report higher levels of somatic symptoms. Qualitative studies could investigate cultural attitudes towards pain and emotional expression among TMD patients, thereby informing culturally sensitive interventions. Similarly, differences in psychosocial stressors between young adults and older individuals may influence somatization patterns. Tailoring interventions to specific subgroups represents a promising direction for future research.

In summary, we provide promising future research directions based on current research gaps about the connection between TMD and somatization. However, as a narrative review, our study is subject to inherent methodological limitations and lacks a standardized methodology, which may introduce selection bias. Moreover, the synthesis and interpretation of findings rely on our judgment, potentially leading to unintended subjectivity despite our efforts to maintain academic rigor.

## 7. Conclusions

In conclusion, we summarize the clinical evidence of the TMD-somatization association, discuss the underlying mechanisms, explore management implications, and identify directions for future research. The robust association between TMD and somatization underscores the importance of a biopsychosocial framework in orofacial pain management. Underlying mechanisms provide a plausible explanation for this association, aligning TMD with other functional somatic syndromes in which the nervous system plays a central role. Health care professionals must recognize the bidirectional influence between TMD pain and somatic distress. Effective care requires interventions that modulate nervous system sensitivity, bolster coping mechanisms, and address local joint pathology concurrently. Continued research is essential to further elucidate this complex relationship and develop targeted therapies.
